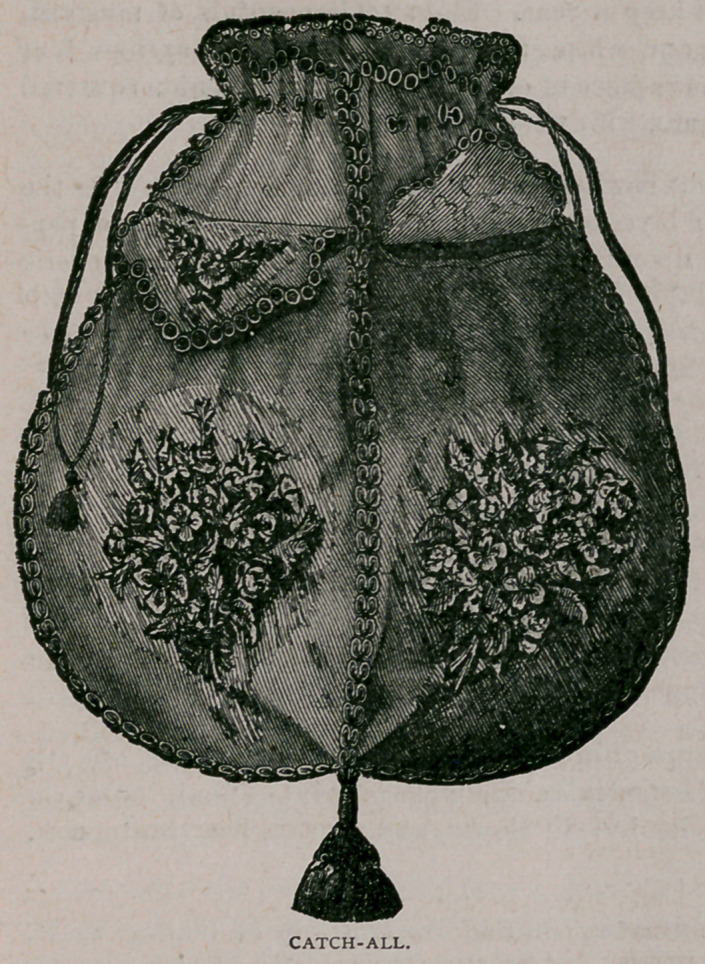# Household

**Published:** 1887-11

**Authors:** 


### HOUSEHOLD.

CATCH-ALL.—The frame-work of this article is made of pieces of card-board sewed together. The materials required for the outside are drab Holland, cretonne flowers, fancy braid, and worsteds to match flowers in color. A cord is drawn through eyelet holes at the top of the bag, and a large tassel of worsted finishes the bottom.

CATCH-ALL.

To take oil spots out of matting etc., wet the spot with alcohol, rub it with hard soap, and then wash well with cold water.

To remove stains from cups or other articles of tableware or marbleized oil-cloths, rub them with saleratus, either with the finger or a piece of linen.

RAGOUT OF BEEF. — One pound and a half of lean beef, two cupfuls of cold water, one finely minced onion. One saltspoonful of allspice, the same of mace. Parsley, thyme and summer savory to taste. Have the meat cut for stewing by the butcher, into pieces about an inch square. Put on the fire in cold water, cover closely and stew slowly for three hours. Half an hour before taking from the fire, add the herbs, onion and other seasoning and thicken with a tablespoonful of browned flour.

MUFFINS.—One egg, one cupful milk, one teaspoonful sugar, two teaspoonfuls yeast powder, and a little salt. Mix with flour not quite so thick as cup cake.

OYSTER FRITTERS.—Make a batter of one coffeecupful milk, one pint flour, one heaping teaspoonful baking powder, two well-beaten eggs and a little salt. Dip the oysters into this batter, and fry singly in hot lard.

OYSTER PIE.—Grease a tin plate and cover the bottom with puffed paste ; lay on it a dozen good-sized oysters and season with butter, pepper and salt ; spread over this an egg batter made of the yelks of two eggs, half a cupful milk, a saltspoonful salt and one cup flour : some prefer a tablespoonful olive oil added. Cover with a crust of the paste, and bake about twenty-five minutes.

ORANGE SAUCE.—Scrape a tablespoonful each of fat bacon and onions; fry them together five minutes, then add the juice of an orange and a tablespoonful of current jelly. Place the sauce pan containing these ingredients where the contents will keep warm, when the baked duck is done, take it up, pour nearly all the fat out of the pan, put in the above mixture, boil up and serve.

GERMAN MUSTARD.—This will keep a year. Eight tablespoonfuls of mustard, four tablespoonfuls each of salt and white sugar, a saltspoonful of cayenne, four tablespoonfuls of melted butter, the juice of one raw onion (a large onion squeezed through a lemon squeezer), and mix with vinegar.

ESCALLOPED OYSTERS.—One pint bowl of rolled crackers ; sprinkle a layer in the bottom of your dish, then put a layer of oysters and sprinkle with salt and pepper, place small pieces of butter upon them, then another layer of rolled crackers, and so on until the dish in full. The top layers should be rolled cracker with pieces of butter on it. Moisten well the whole with milk and water or oyster liquor, bake from an hour to an hour and a half. You will find this very nice.

A DAINTY DESSERT.—Cook six or eight canned pears in their syrup until it becomes like honey, then remove from the fire, halve, and lay in a dish. Beat the whites of two eggs to a stiff froth, sweeten and spread over the pears. Brown in the oven, if desired. This is a nice dessert to serve with the following sponge cake. Three eggs, one and one-half cups sugar, two cups flour, half cup cold water, one teaspoonful soda, and two teaspoonfuls cream tartar. Beat the eggs and sugar until very light, add the soda dissolved in cold water, then the flour in which the cream tartar is sifted, and thoroughly mixed.

CHOCOLATE CARAMELS.—Two cups of brown sugar, one cup of molasses, one cup chocolate grated fine, one cup of boiled milk, one tablespoonful of flour, butter the size of a large English walnut, let it boil slowly, and pour on flat tins to cool, mark off while warm.

CHEAP CAKES.—Two cups of molasses, one and one-half cup of boiling water, two tablespoonfuls (not heaped) of tried-out beef suet, or a piece of butter the size of an egg, 3 heaped teaspoonfuls of baking powder, spices to taste, and a cup of raisins or currants. Add flower enough to make a soft batter, and bake in loaves.

EPICUREAN CAKE.—One cup sugar, yolks of three eggs, two small cups flour, one cup sweet cream. Beat the yolks very light in three tablespoonfuls of sweet milk, mix with the sugar, then add cream and a little salt, sift two teaspoonfuls of baking powder with flour, and mix lightly. Bake in a shallow tin, rather quickly. Flavor to taste. When baked, beat the whites of three eggs to a stiff froth, add one-half cup of white sugar, one teaspoonful of flavoring, and spread over the top, return to the oven a few seconds.

MULLED BUTTERMILK.—To two quarts of fresh buttermilk, when about scalding hot, add a teacup of sugar, two well-beaten eggs, a teaspoonful of ground allspice, and boil together. Have ready in a tureen some nice bits of light bread, and turn the buttermilk over them. Good either hot or cold.

## Figures and Tables

**Figure f1:**